# Identification and Validation of an Immunological Expression-Based Prognostic Signature in Breast Cancer

**DOI:** 10.3389/fgene.2020.00912

**Published:** 2020-09-16

**Authors:** Jianying Pei, Yan Li, Tianxiong Su, Qiaomei Zhang, Xin He, Dan Tao, Yanyun Wang, Manqiu Yuan, Yanping Li

**Affiliations:** ^1^Department of Clinical Laboratory, The First Clinical Medical College of Lanzhou University, Lanzhou, China; ^2^Institute of Clinical Medicine, Gansu Province Maternal and Child-Care Hospital, Lanzhou, China; ^3^Guangdong Provincial Key Laboratory of Stem Cell and Regenerative Medicine, South China Institute for Stem Cell Biology and Regenerative Medicine, Guangzhou Institutes of Biomedicine and Health, Chinese Academy of Sciences, Guangzhou, China; ^4^Department of Clinical Laboratory, People’s Hospital of Qingyang, Qingyang, China; ^5^Department of Hematology, The First Hospital of Lanzhou University, Lanzhou, China; ^6^Department of Clinical Laboratory, The First Hospital of Lanzhou University, Lanzhou, China

**Keywords:** breast cancer, immune-related genes, transcription factors, prognosis, risk-score model

## Abstract

**Background:** Emerging evidence suggests that the immune system plays a crucial role in the regulation of the response to therapy and long-term outcomes of patients with breast cancer (BRCA). In this study, we aimed to identify a significant signature based on immune-related genes to predict the prognosis of BRCA patients.

**Methods:** The expression data were downloaded from The Cancer Genome Atlas (TCGA). The immune-related gene list, the transcription factor (TF) gene list, and the immune infiltrate scores of samples in the TCGA database were acquired from the ImmPort database, the Cistrome Cancer database, and the TIMER database, respectively. Univariate Cox regression analysis was utilized to identify prognostic immune-related differentially expressed genes (DEGs) (PIRDEGs) in BRCA. A prognostic immune signature containing 15 PIRDEGs in BRCA was established using the least absolute shrinkage and selection operator (LASSO) model with 1,000 iterations followed by a stepwise Cox proportional hazards model with a training set of 508 samples in TCGA. An independent assessment of the prognostic prediction ability of the signature was conducted using Kaplan–Meier survival analysis with a testing set of 505 samples in TCGA.

**Results:** We identified 466 PIRDEGs and 80 TFs among the DEGs. A gene signature containing 15 PIRDEGs was constructed. Risk scores of BRCA patients were calculated using this model, which showed a high accuracy of prognosis prediction in both the training set and testing set and could be an independent prognostic factor of BRCA patients.

**Conclusions:** Our study revealed that a PIRDEG signature could be a candidate prognostic biomarker for predicting the overall survival (OS) of patients with BRCA.

## Introduction

As one of the most common malignancies, breast cancer (BRCA) threatens the wellness and health of women worldwide, and the incidence and mortality rates of BRCA are nearly 30 and 15%, respectively (Siegel et al., 2019). Owing to its high heterogeneity, BRCA harbors a plethora of molecular subtypes, which lead to a variety of treatment therapies for BRCA. Hormone exposure is the major risk factor for BRCA, and estrogen receptor (ER), progesterone receptor (PR), and human epidermal growth factor receptor 2 (HER2) are the most common endocrine markers involved in BRCA categorization. Based on the new definition of BRCA molecular subtypes issued by the 2013 St. Gallen International Breast Cancer Conference, BRCA can be classified into the following subtypes: luminal A (ER/PR^+^, HER2^–^, Ki67^+^ < 20%), luminal B (ER/PR^+^ < 20%, HER2^–^, Ki67^+^ ≥ 20%), HER2^+^B2 (ER/PR^+^ and HER2 overexpression), HER2 overexpression (ER^–^, PR^–^, and HER2 overexpression), basal-like TNBC (ER^–^, PR^–^, and HER2^–^), and other subtypes (Goldhirsch et al., 2013). The treatment of BRCA is multidisciplinary and includes local surgery excision, radiation therapy, endocrine therapy, and other chemotherapies, such as anti-HER2 therapy. Consequently, the classification of BRCA subtypes before deciding on a treatment strategy is critical for BRCA patients. For patients with ER^+^ or PR^+^ BRCA, endocrine therapy is considered the most effective way to cure cancer. However, for patients with ER^–^, PR^–^, and HER2^–^ BRCA, traditional endocrine therapy seems to lack efficacy. Thus, screening of the ER or PR state is usually the first step in the clinic toward choosing the treatment method for BRCA patients (Harbeck et al., 2019). In recent years, with the development and application of comprehensive therapies in the clinic, the overall survival (OS) rates for BRCA patients have increased, and the 5-year survival rates have improved to some extent (local stage, >96%; regional stage, >81%; distant stage, >26%) (DeSantis et al., 2017). However, the prognosis of BRCA patients is primarily related to the molecular subtypes, and almost all patients who develop metastatic disease will succumb to it. Thus, it is important to search for novel prognostic biomarkers to predict the response rates to individual therapy of patients with different clinical characteristics or distinct molecular subtypes.

The immune system has been considered a determining factor during cancer initiation and progression (Gentles et al., 2015). Emerging evidence suggests that the immune system plays a crucial role in the regulation of response to therapy and long-term outcomes of patients with BRCA (Savas et al., 2016). Furthermore, oncology and immunology are interwoven, especially in the selection of tumor therapy and prognosis prediction. Thus, the development of oncoimmunology has revealed that tumor-infiltrating lymphocytes (TILs), which are immune cells that infiltrate tumor tissues and increase the expression of immune-related genes, are closely related to the better survival of patients with specific subtypes of BRCA (Gooden et al., 2011; Karn et al., 2011). TILs have been declared in various types of solid tumors, including BRCA, colon cancer, melanoma, cervical cancer, and lung cancer (Underwood, 1974; Savas et al., 2016). Elevated levels of lymphocytic infiltrate were reported to be associated with HER2 amplification and portended long-term clinical outcomes (Tang et al., 1990). A strong linear relationship between the TIL number in patients with triple-negative breast cancer and the recurrence-free survival endpoints that increased over time has been reported (Loi et al., 2013). A previous study showed that high infiltration of lymphocytes in BRCA tissues might predict the response to neoadjuvant therapy and may also have a significant prognostic value after adjuvant chemotherapy (Denkert et al., 2016). An increasing number of reports have confirmed that immune cells and immune-related genes have significant prognostic and predictive values. However, little is known about the genomic features driving high or low immune infiltration in BRCA; thus, it is of great value to better understand the interaction of BRCA and the immune system and to discover more potential immuno-oncological prognostic and predictive markers.

Owing to technique limitations, an accurate assessment of TILs has not been achieved; for instance, tissue-based methods, including flow cytometry and immunohistochemistry, cannot be applied to the high-throughput examination of multiple markers and large number of samples simultaneously. Progress in the field of single-cell genomes and transcriptomes has provided us with gene expression data to assess immune-related classifications and their associations with tumor cells (Charoentong et al., 2017; Liu and Mardis, 2017). The development of bioinformatics techniques enables the integration of gene expression data into biological data; numerous emerging databases offer large-scale data with gene expression, clinical information, and biological characteristics; and various bioinformatics tools provide opportunities to merge different resources derived from different studies. For example, Li et al. (2016) created the web resource database TIMER to evaluate the clinical impact of immune cells in diverse cancers. The ImmPort database was put in place to function as a critical repository for immunology-related clinical and molecular data (Bhattacharya et al., 2014). With all these approaches, researchers have begun to quantify TILs using the expression values of immune-related genes and identify individualized immune-related signatures in the tumor prognosis of various types of cancers, including melanoma, lung cancer, glioblastoma, and BRCA (Cheng et al., 2016; Li et al., 2019; Song et al., 2019; Yang et al., 2020). Cheng et al. (2016) identified eight genes (FOXO3, IL6, IL10, ZBTB16, CCL18, AIMP1, FCGR2B, and MMP9) with the highest prognostic value in glioblastoma using The Cancer Genome Atlas (TCGA) database. Li et al. (2019) identified four immune-related genes (APOD, CXCL14, IL33, and LIFR) correlated with BRCA prognosis.

**FIGURE 1:**
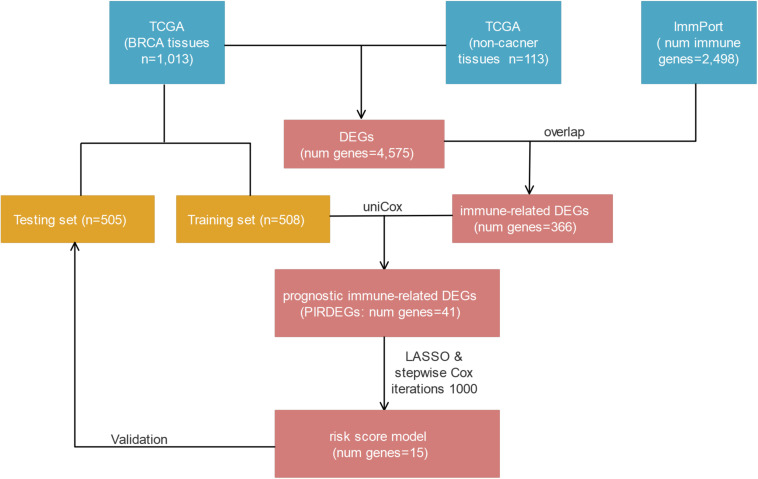
The workflow of risk-score model construction. The blue, yellow, and red cells indicate the corresponding datasets or gene lists.

In this study, we focused on investigating the role of immune-related genes that are differentially expressed in BRCA tissues compared with healthy tissues and on exploring a model composed of immune-related genes to predict the prognosis of patients with BRCA. With this goal, we combined multiperspective databases, such as TCGA, ImmPort, TIMER, and Cistrome, with multidimensional analysis methods, such as differential analysis, univariate or multivariate Cox analysis, risk-score model construction, survival analysis, receiver operating characteristic (ROC) curve analysis, prognosis verification, and correlation analysis. As a result, we found a prognostic risk-score model for BRCA patients who contained 15 prognostic immune-related differentially expressed genes (DEGs) (PIRDEGs) in BRCA. We constructed a regulatory network of transcription factors (TFs) and IRDEGs in BRCA. We verified the application value of the risk-score model and analyzed the correlation between the risk score and the number of immune cells in BRCA tissues. Overall, our study discovered a prognostic signature model with relatively high application potential in BRCA. The workflow is shown in Figure 1.

## Results

### Identification of Differentially Expressed Genes and Prognostic Immune-Related Differentially Expressed Genes in Breast Cancer

**FIGURE 2:**
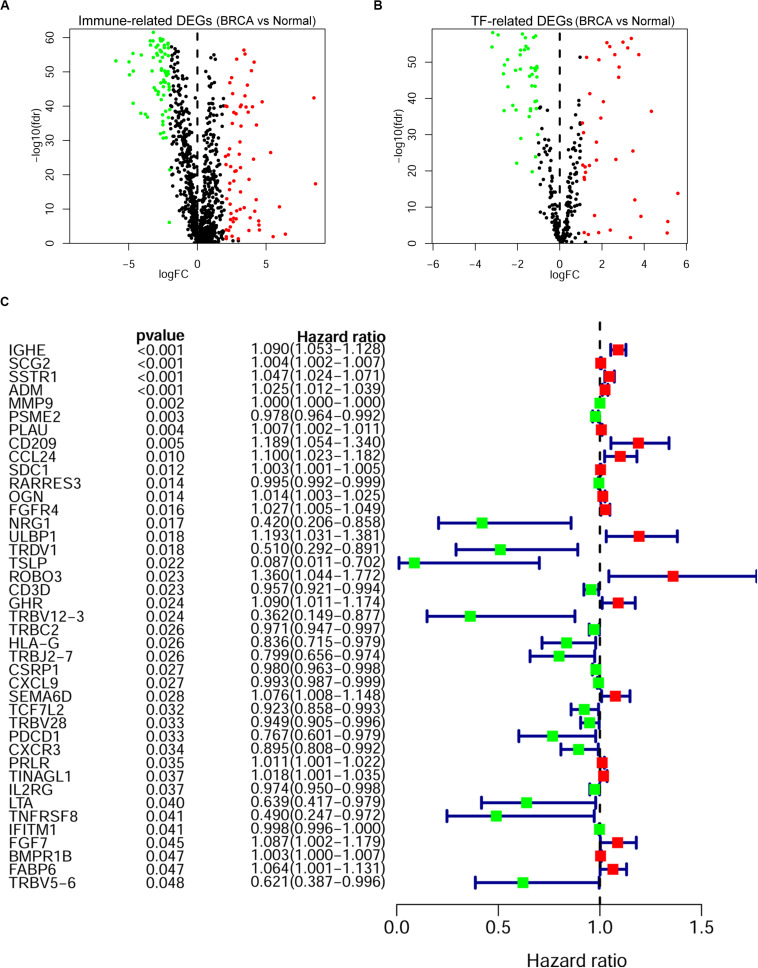
Screening of the prognostic immune-related differentially expressed genes (DEGs) in breast cancer (BRCA). The volcano plot of immune-related DEGs **(A)** and transcription factor (TF)-related DEGs **(B)** in BRCA. Each plot indicates a gene; genes with | log2-fold change| < 1 or false discovery rate (fdr) > 0.05 are shown in black; genes with log2-fold change > 1 and fdr < 0.05 are shown in red; and genes with log2-fold change < -1 and fdr < 0.05 are shown in green. **(A)** A total of 366 immune-related DEGs, 193 of which were upregulated (red) and 173 of which were downregulated (green), were identified. **(B)** Eighty TF-related DEGs: 38 were upregulated (red) and 42 were downregulated (green). **(C)** Forest plot of gene expression and prognosis of BRCA patients. Univariate Cox analysis identified 41 immune-related DEGs correlated with the overall survival of BRCA patients; red cells indicate high-risk genes, and green cells indicate low-risk genes.

A cohort of 1,222 samples, consisting of 1,109 BRCA patients and 113 normal individuals from the TCGA database, was used to identify the DEGs between BRCA tissues and normal tissues. We downloaded the RNA-Seq data from TCGA and genes that met the criteria: |log2-fold change| > 1 and false discovery rate (fdr) < 0.05 were defined as DEGs. As shown in Supplementary Table 1, we finally identified 4,575 DEGs, of which 2,698 were upregulated and 1,877 were downregulated (Supplementary Figures 1A,B). Then, to identify the immune-related genes that play a regulatory role in BRCA, we searched and downloaded all 2,498 immune-related genes from the immunological database ImmPort (Supplementary Table 2). We matched these immune-related genes with DEGs in BRCA, and then we obtained 366 immune-related genes that also belonged to the set of DEGs in BRCA and their detailed expression pattern (Figure 2A and Supplementary Figure 2A and Supplementary Table 3).

### The Regulatory Network Between Transcription Factors and Prognostic Immune-Related Differentially Expressed Genes

**FIGURE 3:**
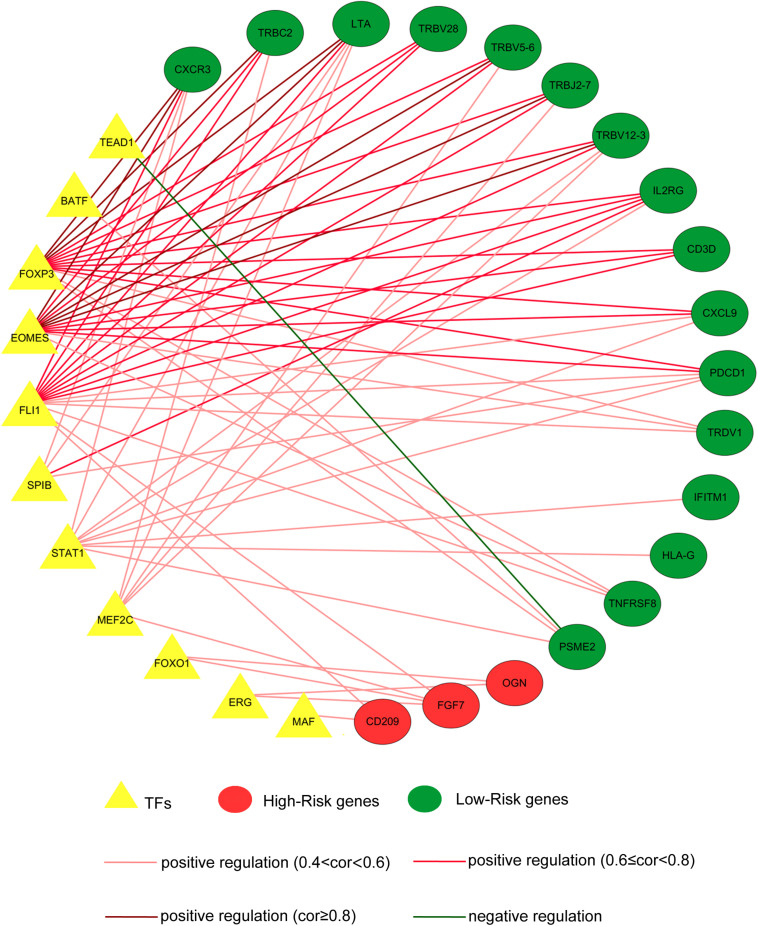
The transcription factor–prognostic immune-related differentially expressed gene (TF–PIRDEG) regulatory network in breast cancer (BRCA). The red circles indicate high-risk genes, and the green circles indicate low-risk genes. The yellow triangles indicate the TFs, and the green line indicates a negative correlation. The light red lines, red lines, and dark red lines indicate positive correlations with correlation coefficients of 0.4–0.6, 0.6–0.8, and higher than 0.8, respectively.

As key factors regulating gene expression, TFs function as gene transcription accelerators or inhibitors by modulating the transcription of downstream genes. To reveal the crucial mechanisms underlying the transcriptional regulation of immune-related genes in BRCA, we constructed a transcriptional regulatory network of immune-related genes in BRCA. First, 318 tumor-related TFs were downloaded from the Cistrome Cancer database and were matched with the identified DEGs in BRCA (Supplementary Table 4). There were 80 tumor-related TFs among the DEGs (Figure 2B and Supplementary Table 5 and Supplementary Figure 2B). To construct a model, we randomly classified the 1,013 BRCA samples in TCGA (96 samples were excluded owing to the lack of enough clinical information) into two groups: a training set (*n* = 508) and a testing set (*n* = 505). To discover which immune-related genes affected the prognosis of BRCA patients, we subjected the expression values of all 366 immune-related DEGs to a univariate Cox proportional hazards regression analysis in the training set and identified 41 PIRDEGs that strongly correlated with patient OS (*p* < 0.05). Twenty of the 41 PIRDEGs were high-risk genes, and the other 21 PIRDEGs were low-risk genes for OS in BRCA patients (Figure 2C). All 41 PIRDEGs and 80 TFs among DEGs were used to construct the transcriptional regulatory network of PIRDEGs via Pearson’s correlation analysis. To find strong correlations between TFs and PIRDEGs, we set the correlation analysis parameter filters as |correlation coefficient >0.4| and *p* < 0.001. As shown in Figure 3 and Supplementary Tables 6, 11 TFs among DEGs and 19 PIRDEGs constituted the transcriptional regulatory network of PIRDEGs. In this network, almost all of the TFs played a positive regulatory role, and only the regulatory relationship between TEAD1 and PSME2 was negative. In this network, the correlation degree was indicated using the color of the edge lines: the light red lines indicated that the correlation coefficient was between 0.4 and 0.6; the red lines indicated that the correlation coefficient was between 0.6 and 0.8; and the dark red lines indicated a correlation coefficient higher than 0.8.

### An Immune Gene Signature Model Predicted the Overall Survival of Breast Cancer Patients

**FIGURE 4:**
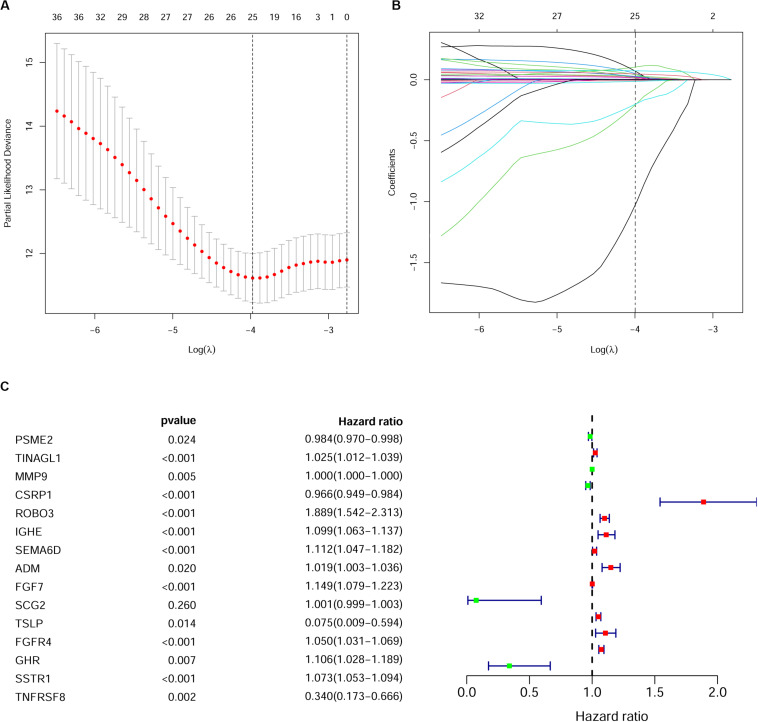
Establishment of the immune gene signature. **(A)** A 1,000-fold cross-validation for tuning parameter selection in the least absolute shrinkage and selection operator (LASSO) model. Partial likelihood deviance is shown as the mean ± standard deviation. Numbers in the top margin indicate the gene numbers included in each LASSO model. **(B)** LASSO coefficient profiles of the most useful prognostic genes. Numbers in the top margin indicate the gene numbers included in each LASSO model. Each line indicates an individual gene in the LASSO model. **(C)** Forest plot of immune genes in the model in the training set.

As described above, we identified 41 PIRDEGs using univariable Cox regression analysis. We used the least absolute shrinkage and selection operator (LASSO) Cox regression model to select the most useful model, and a gene model above the minimum deviance with 25 genes was identified (Figures 4A,B and Supplementary Table 7). Next, we further performed a stepwise Cox proportional hazards regression model and finally obtained a model consisting of 15 PIRDEGs – PSME2, TINAGL1, MMP9, CSRP1, ROBO3, IGHE, SEMA6D, ADM, FGF7, SCG2, TSLP, FGFR4, GHR, SSTR1, and TNFRSF8 (Figure 4C and Table 1) – among which PSME2, CSRP1, TSLP, and TNFRSF8 with negative coefficients were low-risk PIRDEGs and TINAGL1, MMP9, ROBO3, IGHE, SEMA6D, ADM, FGF7, SCG2, FGFR4, GHR, and SSTR1 with positive coefficients were high-risk PIRDEGs. The high-risk IRDEGs negatively correlated with the prognosis of BRCA patients, while the low-risk IRDEGs positively correlated with the prognosis of BRCA patients. Subsequently, a formula composed of the expression values and coefficients of genes in this model was chosen to calculate the risk score of each sample as follows: risk score = (−0.01612 × expression value of PSME2) + (0.025082 × expression value of TINAGL1) + (0.000253 × expression value of MMP9) + (−0.03458 × expression value of CSRP1) + (0.635804 × expression value of ROBO3) + (0.094539 × expression value of IGHE) + (0.106281 × expression value of SEMA6D) + (0.019012 × expression value of ADM) + (0.138697 × expression value of FGF7) + (0.001191 × expression value of SCG2) + (−2.59049 × expression value of TSLP) + (0.048535 × expression value of FGFR4) + (0.100568 × expression value of GHR) + (0.070616 × expression value of SSTR1) + (−1.07935 × expression value of TNFRSF8). On the basis of this formula, we obtained the risk score of each sample in the training set and the testing set (Supplementary Table 8).

**TABLE 1:** The immune-genes signature model.

ID	Coeff	HR	HR.95L	HR.95H	*p*-value
PSME2	−0.01612	0.984011	0.970359	0.997856	0.023753
TINAGL1	0.025082	1.025399	1.011599	1.039387	0.000286
MMP9	0.000253	1.000253	1.000076	1.000429	0.005004
CSRP1	−0.03458	0.966014	0.948765	0.983576	0.000169
R0B03	0.635804	1.888541	1.541644	2.313495	8.25E-10
IGHE	0.094539	1.099152	1.062536	1.137029	4.52E-08
SEMA6D	0.106281	1.112134	1.046724	1.181632	0.000589
ADM	0.019012	1.019194	1.002936	1.035716	0.020491
FGF7	0.138697	1.148776	1.079188	1.222851	1.36E-05
SCG2	0.001191	1.001191	0.999118	1.003269	0.26023
TSLP	−2.59049	0.074983	0.009463	0.594155	0.01417
FGFR4	0.048535	1.049732	1.030649	1.069168	2.16E-07
GHR	0.100568	1.105799	1.028374	1.189053	0.00662
SSTR1	0.070616	1.073169	1.052606	1.094133	8.43E-13
TNFRSF8	−1.07935	0.339816	0.173307	0.666304	0.001679

### Validation of the Immune Gene Signature for Survival Prediction

**FIGURE 5:**
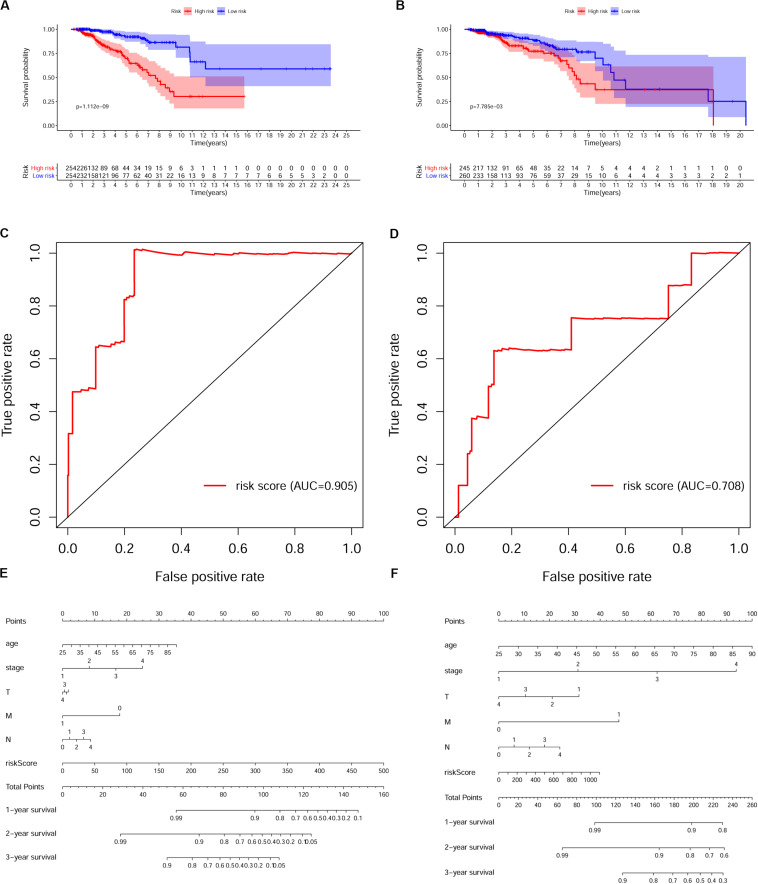
Validation of the prognostic risk model in breast cancer (BRCA) patients. **(A,B)** Kaplan–Meier survival analysis of overall survival (OS) in the high-risk (red) and low-risk (blue) patients with BRCA in the training set. The colors of each survival line indicate the 95% CI of probability of survival at each time point **(A)** and testing set **(B)**. Log-rank test, *p* < 0.05. **(C,D)** The receiver operating characteristic (ROC) curve shows the area under the ROC curve (AUC) value of the risk model in the training set **(C)** and testing set **(D)**. **(E,F)** Nomograms of the risk-score model in the training set **(E)** and testing set **(F)**.

**FIGURE 6:**
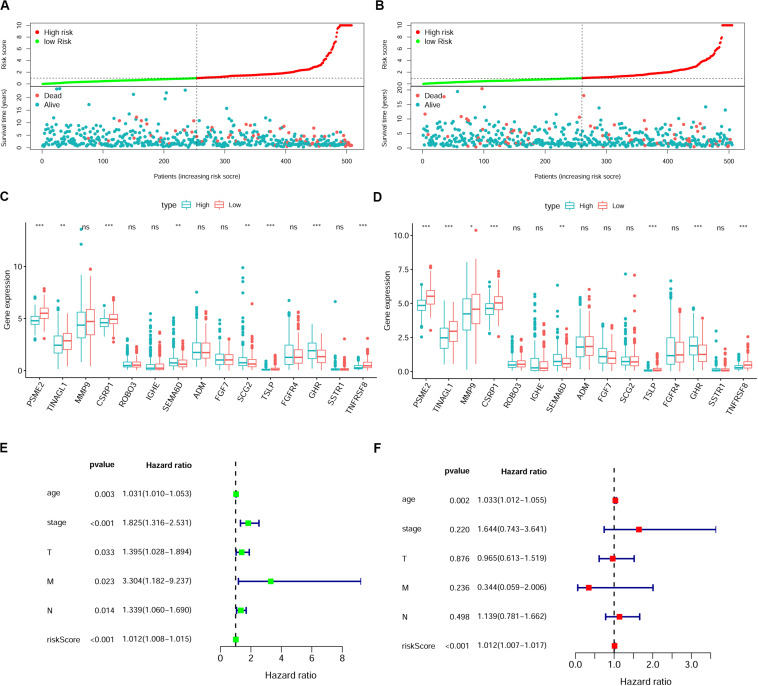
The immune gene signature is an independent prognostic indicator in breast cancer (BRCA). **(A,B)** Risk-score distribution and survival status of BRCA patients in the training set **(A)** and testing set **(B)**. **(C,D)** Expression of risk genes in the high-risk or low-risk group of BRCA patients in the training set **(C)** and testing set **(D)**, Student’s *t* test. **(E,F)** Univariate **(E)** and multivariate **(F)** Cox analyses of clinical characteristics or risk scores with the prognosis of patients with BRCA in the training set. T, tumor depth; N, lymph node invasion; M, distant metastasis.

To verify whether this risk-score model could precisely predict the prognosis of BRCA patients, we utilized the training set and testing set to validate the prognosis prediction ability of this model. The cutoff value of the risk score was the median risk score in the training set. On the basis of this parameter, we divided all samples in the training set and the testing set into high-risk groups or low-risk groups. Kaplan–Meier survival analysis was performed. As expected, the high-risk group showed a poorer OS rate than the low-risk group in both the training set and testing set (Figures 5A,B). Next, we examined the predictive performance of this risk-score model for OS using ROC curves, and the results showed that the areas under the ROC curve (AUCs) in the training set and the testing set were 0.905 and 0.708, respectively (Figures 5C,D). The nomograms of this model in the training set and the testing set are shown in Figures 5E,F, respectively. We then ranked the risk scores of patients and analyzed the distribution of OS status of each patient in the training set and the testing set. As shown in Figures 6A,B, in the upper panel, the red dot plots indicate the high-risk patients, while the green dot plots indicate the low-risk patients; in the lower panel, the pink dot plots indicate patients who were dead, and the cyan dot plots indicate patients who were alive. It was clear that, in the lower panel, the number of pink dot plots increased with the rise of risk scores of patients. The bar plots show the expression pattern of risk genes in patients in the high- and low-risk groups (Figures 6C,D). We performed univariate and multivariate Cox regression analyses to examine whether the immune gene signature was an independent prognostic factor in BRCA patients. We first analyzed the correlations between clinical factors, including age, gender, TNM stage, or risk scores, and OS of BRCA patients in the training group. In the univariate Cox analysis, age, TNM stage, and risk score were independent prognostic factors of BRCA patients (*p* < 0.05) (Figure 6E). Survival outcomes of tumor patients can be affected by multiple factors; thus, we defined all these clinical features as covariates and performed multivariate Cox analysis. As shown in Figure 6F, the prognostic prediction power of the 11 immune gene signatures was independent of the clinical features (HR = 1.012, 95% CI: 1.007–1.017, *p* < 0.001).

### The Associations of the Immune Gene Signature and Clinical Characteristics in Breast Cancer

**FIGURE 7:**
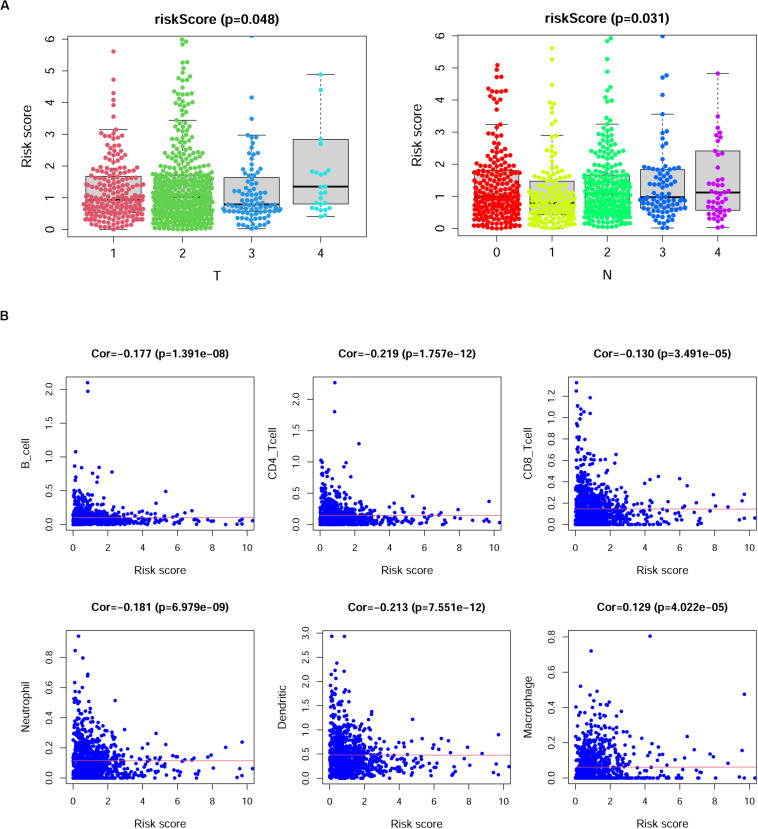
The correlation between risk scores and clinical characteristics of breast cancer (BRCA) patients. **(A)** Risk scores showed differences among different tumor depth groups and lymph node invasion groups. T, tumor depth; N, lymph node invasion. Kruskal–Wallis test. **(B)** The correlation between risk scores and the numbers of six types of immune cells in patients with BRCA. Cor., Spearman correlation coefficient.

We then investigated the correlations between the risk scores derived from this model and the clinical characteristics in BRCA patients. The results indicated that risk scores in the T4 group were higher than those in the T1, T2, and T3 groups (*p* = 0.048); however, we observed that the sample number of the T4 group was smaller than that of the other groups; thus, this result may need further verification. Additionally, the risk scores showed a gradual increase with increasing lymph node invasion degree (Figure 7A). We also analyzed the correlation between the specific expression level of immune genes in this model and the clinical characteristics of BRCA (Supplementary Figure 3).

### The Associations of the Immune Gene Signature and Immune Cell Infiltration

Previous studies reported that immune cell infiltration levels were associated with favorable outcomes of patients (Cheng et al., 2016; Li et al., 2019; Song et al., 2019; Yang et al., 2020). Thus, we analyzed the correlation of risk scores and the numbers of six infiltrating immune cell types in patients with BRCA, namely, B cells, CD4^+^ T cells, CD8^+^ T cells, neutrophils, dendritic cells, and macrophages. As shown in Figure 7B, the risk scores of BRCA patients displayed negative correlations with the infiltration of B cells, CD4^+^ T cells, CD8^+^ T cells, neutrophils, and dendritic cells in BRCA tissues, while the risk scores of BRCA patients displayed positive correlations with the infiltration of macrophages (*p* < 0.05).

## Discussion

Immune system dysregulation has been documented in many types of cancers. Investigators have reported the activation of immune-related genes in BRCA (Ascierto et al., 2012). An increasing number of studies have emphasized the significance of prognostic biomarkers in predicting cancer outcomes and recommended that more investigators include these biomarkers into therapeutic research (Li et al., 2016). Several studies have suggested that immune-related genes can be used as prognostic biomarkers in BRCA (Gingras et al., 2015; Li et al., 2019). In the past years, most studies about immune-related prognostic biomarkers focused on TILs, and TILs have been identified as prognostic biomarkers in multiple malignancies. High infiltration of CD45RO^+^ (memory) and CD8^+^ (cytotoxic) TILs (used to calculate the immunoscore) was strongly related to the clinical outcomes of patients with lung, breast, colon, ovarian, prostate, and head and neck cancers (Vano et al., 2018). For instance, it has been reported that the densities and type of CD8^+^ TILs were correlated with tumor progression without interference from the tumor stage in colorectal cancer. Thus, the immunoscore was considered a valuable prognostic factor (Baxevanis et al., 2019). The core of TILs is immune cells, and related studies have focused on the cell level. However, the definition of cell type, especially an immune cell type, is still dependent on molecular protein markers. Thus, we proposed as the original aim of our study to develop a series of immune-related biomarkers composed of immune genes rather than immune cells. In our study, to identify the molecular biomarkers in BRCA, we first identified the DEGs between BRCA and healthy tissues using public datasets from TCGA. Then, our study aimed to develop immune-related biomarkers; thus, the immune-related database ImmPort was used to identify the immune genes and help us obtain the IRDEGs in BRCA. PIRDEGs were selected using univariate Cox analysis.

Transcription factors play a crucial role in controlling the expression of genes by binding to a particular DNA region, such as an enhancer or promoter. TFs were also found to be associated with the progression of glioblastoma (Wei et al., 2015). To construct a regulatory network of immune genes in BRCA, we selected 80 TFs from the DEGs in BRCA on the basis of information from the Cistrome database. Then, we constructed the regulatory network of TFs-PIRDEGs in BRCA on the basis of their correlations. As shown in Figure 3 and Supplementary Table 6, we found four key TFs – EOMES, FOXP3, FLI1, and STAT1 – which had the most downstream PIRDEGs and relatively high correlation coefficients. Among the 41 PIRDEGs in this regulatory network, 21 PIRDEGs were the downstream genes of EOMES, and the correlation coefficient between EOMES and CXCR3 was up to 0.86. EOMES has been demonstrated to be involved in the differentiation of CD8^+^ T cells during the immune response by regulating the expression of lytic effector genes (Atreya et al., 2007) and to be an independent good prognostic factor for progression-free survival and OS, likely due to its association with a favorable immune signature in metastatic renal cell cancer patients (Dielmann et al., 2016). FOXP3 is an essential TF in maintaining immune system hemostasis by regulating Treg lineage function, and it has been described to be a strong prognostic factor for distant metastasis-free survival of BRCA patients (Merlo et al., 2009). In the TF-PIRDEG network, we found many T-cell receptor-coding genes, including T-cell receptor beta variable and constant genes (TRBV5-6, TRBV28, TRBV18, TRBV12-3, and TRBC2).

We constructed a prognostic risk-score model using LASSO Cox regression analysis and obtained an immune gene signature to predict the OS of BRCA patients. As shown in Table 1, four low-risk IRDEGs and 11 high-risk IRDEGs constituted this risk-score model, and each patient was assigned a risk score based on this model. One of the immune genes in the signature and a high-risk factor in this model, FGF7, had the highest coefficient at 0.14. FGF7 is secreted by mesenchymal origin cells and acts as a paracrine cytokine targeting nearby cells via locally secreted signals, thus participating in immune reactions during tumor progression or inhibition (Finch and Rubin, 2006). In urothelial carcinoma of the upper urinary tract and bladder, FGF7 overexpression is an independent prognosticator (Fan et al., 2015). TSLP, TINAGL1, TNFRSF8, and PSME2 in this model exerted negative correlations with the OS of BRCA patients. TSLP is a member of cytokine resembling cytokine IL-7 and has recently been reported to be a regulator of type 2 inflammatory responses (Sims et al., 2000). The function of TSLP in various types of tumors is tumor context dependent, and its role in BRCA is also controversial. One study revealed that an IL-1β–TSLP pathway could blunt antitumor immunity. In contrast, another study proposed its pro-tumor role with the evidence that the keratinocyte-specific overexpression of TSLP (in K14-TSLP transgenic mice) could inhibit the development of early BRCA (Corren and Ziegler, 2019). In our study, TSLP showed a protective effect on the OS of BRCA patients. PSME2 is a subunit of proteasome activator PA28 participating in the generation of class I binding peptides such as antigens from viruses or tumors (Sijts et al., 2002).

All these prognostic signatures identified in our study are immune system regulators and play a significant role in the cellular immune reaction under different situations. By combining them, we obtained a prognosis prediction model. The efficacy of this model was verified in the training set and the testing set simultaneously, the OS rates of high- and low-risk-score groups of BRCA patients showed a significant difference, and the number of dead BRCA patients increased with the increase in risk scores of BRCA patients. Both univariate and multivariate Cox analyses revealed that the risk score derived from this model was an independent prognostic factor for BRCA patients. Early studies identified TILs in BRCA that mainly comprised CD8^+^ T cells, CD4^+^ T cells, CD19^+^ B cells, and rare NK cells (Savas et al., 2016). In our study, we found that the risk scores showed negative correlations with the numbers of B cells, CD8^+^ T cells, CD4^+^ T cells, and neutrophil cells. The prognostic value of TILs is somewhat debatable; for instance, in most human cancers, increased densities of CD3^+^, CD8^+^, and memory CD45RO^+^ T cells are associated with favorable prognosis. However, the prognostic value of these cells may be influenced by other immune cells residing in the same tumor (Baxevanis et al., 2019). Hence, we may conclude that infiltrating immune cells as prognostic biomarkers in tumor patients are, to a large extent, dependent on the comprehensive assessment of various types of immune cells.

In our study, we focused on the gene level rather than the cell level by screening PIRDEGs using bioinformatics analysis in BRCA, and the TF-PIRDEG network was used to identify crucial TFs in the regulation of the transcription level of immune-related genes in BRCA. A risk-score model consisting of immune genes was built, and the efficacy of this model was examined using extensive data from real samples. However, we cannot exclude the possibility that other factors, such as age and sex, may introduce bias in the practical values of the identified genes. In addition, the data types and the random classification of the training group and test group may lead to different models. For instance, the public database TCGA offers researchers various data types, including raw gene counts, FPKM and FPKM-UQ. We used the FPKM files in our study, and the other two file types can also be used for analysis. In our study, we randomly evenly classified the TCGA samples into two groups, which is not the only way to categorize. We considered the different genes and prognostic models that may be found in other studies. Additionally, the different construction methods of the prognostic model and various algorithms that were used during the model construction may also lead to other models harboring equal or higher prediction value. Furthermore, our study was completely dependent on the reanalysis of public data, which was appropriate but lacked validation using clinical samples. We thought it would be more convincing if we added another test group using independent clinical samples, such as examination of the expression at the transcriptional level of the identified genes in our model using RT-PCR or Western blot. Then, validating the regulatory network of TFs and these genes and the molecular mechanisms of tumor immunity may also provide investigators with more accurate and convincing evidence for future application of such regulatory networks in the clinic.

## Materials and Methods

### Data Collection and Preprocessing

The transcriptome profiles (HTSeq-FPKM) of 1,222 samples, consisting of 1,109 breast tissues of patients with BRCA and 113 adjacent tissues, and corresponding clinical information were downloaded from the TCGA database in December 2019^1^. Expression data or clinical information of each sample were combined into corresponding matrix files using Perl language^2^. Ensemble IDs in the expression matrix profile were also converted into Gene Symbols with Perl language. ID numbers in the expression profile and clinical information profile were matched, and samples whose ID numbers did not match were excluded from our study. Finally, we obtained 1,097 BRCA cases for subsequent analysis. Immune-related genes were acquired from the ImmPort database^3^. Tumor-related TFs were obtained from the Cistrome Cancer database^4^. The immune infiltrate scores of samples in the TCGA database were obtained from the TIMER database^5^.

### Screening of Immune-Related or Transcription Factor-Related Differentially Expressed Genes

The RNA-Seq profiles from TCGA were transformed using log2, and the DEGs were identified using the limma package in R software. The parameters for DEG selection were as follows: |log2-fold change| > 1 and false discovery rate <0.05. Volcano plots and heatmaps were drawn using the limma or pheatmap package in R. The genes overlapping between DEGs and immune-related genes were defined as immune-related DEGs. The genes overlapping between DEGs and tumor-related TFs were defined as TF DEGs.

### Survival Analysis and Risk-Score Model Establishment Using Cox Regression Analysis

A total of 366 immune-related DEGs were subjected to univariate Cox proportional hazard regression analysis to identify PIRDEGs. PIRDEGs with statistical significance in the univariate Cox regression analysis were tested in the LASSO Cox regression analysis to generate a coefficient of each immune-related DEG. For the Cox analysis, the cox.zph function in the survival package in R was used to guarantee that the proportional hazards assumption is appropriate. A risk-score model was built using the formula *Risk Score* $\left(RS\right)=\sum_{i=1}^{n}\left(Expi^{*}Coei\right)$, where *n* is the number of IRDEGs; *Expi* is the expression value of each PIRDEG; and *Coei* is the estimated regression coefficient of each PIRDEG derived from the multivariate Cox regression analysis. Based on this formula, each patient can be assigned a risk score, which is a linear combination of the expression value of the selected PIRDEGs weighted by their coefficients.

### Construction of the Transcription Factor–Prognostic Immune-Related Differentially Expressed Gene Regulatory Network

The coexpression relationship between TF DEGs and PIRDEGs was analyzed using the corrplot package in R software, and Pearson’s correlation was calculated. The selection criteria of the correlation coefficient were as follows: |cor| > 0.4, *p* < 0.001. The network was visualized using Cytoscape software.

### Value Assessment of the Risk-Score Model

Relying on the risk score of each patient from the RS formula, we first selected the median risk score of the training set as the cutoff and divided all BRCA patients of the training set or testing set into two groups: the high-risk group and the low-risk group. The survival analysis between these two groups was conducted using the survival package in R software. The accuracy of our risk-score model to predict prognosis was evaluated using the ROC curve, and the ROC curve was calculated and generated using the survivalROC package in R software. Univariate or multivariate Cox analyses were used to test whether the risk score of our model is an independent prognostic factor in BRCA. Cox analysis was performed, and forest graphs were generated using the survival package in R.

### Correlation Analysis of Clinical and Immune Characteristics

The correlations between risk scores or gene expression value in the risk-score model and clinical characteristics, including tumor stages, tumor grades, and lymph node metastasis, were performed using the beeswarm package in R. The correlation between risk scores and immune infiltrate scores was assessed using the corrplot package in R software.

### Statistical Analysis

All statistical analyses were performed using R software. The Wilcox test in R was applied to identify differences between the normal group and the tumor group. The two-sided log-rank test in the survival package in R was employed to assess the survival difference between the high-risk and low-risk groups, and DEG multivariate analyses were conducted using the Cox proportional hazards regression model. *p* < 0.05 was considered statistically significant.

## Data Availability Statement

The datasets generated for this study can be found in the TCGA database (https://portal.gdc.cancer.gov), ImmPort database (http://www.immport.org), Cistrome Cancer database (http://cistrome.org/CistromeCancer/), and TIMER database (http://cistrome.dfci.harvard.edu/TIMER/).

## Author Contributions

JP and YL designed the study and performed part of the data analysis. TS, QZ, and XH collected data and performed the literature search. DT and YW performed part of the data analysis and revised the images. MY wrote the manuscript. YpL revised the manuscript. All authors contributed to the article and approved the submitted version.

## Conflict of Interest

The authors declare that the research was conducted in the absence of any commercial or financial relationships that could be construed as a potential conflict of interest.

## Footnotes

1 https://portal.gdc.cancer.gov

2 http://www.perl.org/

3 http://www.immport.org

4 http://cistrome.org/CistromeCancer/

5 http://cistrome.dfci.harvard.edu/TIMER/

## Supplementary Material

FIGURE S1 Heatmap **(A)** and volcano plot **(B)** of DEGs in BRCA.

FIGURE S2 Heatmap of immune-related genes **(A)** and TFs **(B)** in DEGs in BRCA.

FIGURE S3 The correlation between expression levels of immune genes in the risk score model and clinical characteristics of BRCA patients.

## Abbreviations

AUC: area under the curve
BRCA: breast cancer
DEGs: differentially expressed genes
ID: identity
OS: overall survival
PIRDEGs: prognostic immune-related differentially expressed genes
ROC: receiver operating characteristic curve
TCGA: The Cancer Genome Atlas
TF: transcription factor
TILs: tumor-infiltrating lymphocytes.

## References

[B1] Ascierto M. L. Kmieciak M. Idowu M. O. Manjili R. Zhao Y. Grimes M. (2012). A signature of immune function genes associated with recurrence-free survival in breast cancer patients. *Breast Cancer Res. Treat.* 131 871–880. 10.1007/s10549-011-1470-x 21479927 PMC3431022

[B2] Atreya I. Schimanski C. C. Becker C. Wirtz S. Dornhoff H. Schnurer E. (2007). The T-box transcription factor eomesodermin controls CD8 T cell activity and lymph node metastasis in human colorectal cancer. *Gut* 56 1572–1578. 10.1136/gut.2006.117812 17566017 PMC2095672

[B3] Baxevanis C. N. Sofopoulos M. Fortis S. P. Perez S. A. (2019). The role of immune infiltrates as prognostic biomarkers in patients with breast cancer. *Cancer Immunol. Immunother.* 68 1671–1680. 10.1007/s00262-019-02327-7 30905043 PMC11028310

[B4] Bhattacharya S. Andorf S. Gomes L. Dunn P. Schaefer H. Pontius J. (2014). ImmPort: disseminating data to the public for the future of immunology. *Immunol. Res.* 58 234–239. 10.1007/s12026-014-8516-1 24791905

[B5] Charoentong P. Finotello F. Angelova M. Mayer C. Efremova M. Rieder D. (2017). Pan-cancer immunogenomic analyses reveal genotype-immunophenotype relationships and predictors of response to checkpoint blockade. *Cell Rep.* 18 248–262. 10.1016/j.celrep.2016.12.019 28052254

[B6] Cheng W. Ren X. Zhang C. Cai J. Liu Y. Han S. (2016). Bioinformatic profiling identifies an immune-related risk signature for glioblastoma. *Neurology* 86 2226–2234. 10.1212/wnl.0000000000002770 27225222

[B7] Corren J. Ziegler S. F. (2019). TSLP: from allergy to cancer. *Nat. Immunol.* 20 1603–1609. 10.1038/s41590-019-0524-9 31745338

[B8] Denkert C. Wienert S. Poterie A. Loibl S. Budczies J. Badve S. (2016). Standardized evaluation of tumor-infiltrating lymphocytes in breast cancer: results of the ring studies of the international immuno-oncology biomarker working group. *Mod. Pathol.* 29 1155–1164. 27363491 10.1038/modpathol.2016.109

[B9] DeSantis C. E. Ma J. Goding Sauer A. Newman L. A. Jemal A. (2017). Breast cancer statistics, 2017, racial disparity in mortality by state. *CA Cancer J. Clin.* 67 439–448. 10.3322/caac.21412 28972651

[B10] Dielmann A. Letsch A. Nonnenmacher A. Miller K. Keilholz U. Busse A. (2016). Favorable prognostic influence of T-box transcription factor eomesodermin in metastatic renal cell cancer patients. *Cancer Immunol. Immunother.* 65 181–192. 10.1007/s00262-015-1786-1 26753694 PMC11029520

[B11] Fan E. W. Li C. C. Wu W. J. Huang C. N. Li W. M. Ke H. L. (2015). FGF7 Over expression is an independent prognosticator in patients with urothelial carcinoma of the upper urinary tract and bladder. *J. Urol.* 194 223–229. 10.1016/j.juro.2015.01.073 25623741

[B12] Finch P. W. Rubin J. S. (2006). Keratinocyte growth factor expression and activity in cancer: implications for use in patients with solid tumors. *J. Natl. Cancer Inst.* 98 812–824. 10.1093/jnci/djj228 16788155

[B13] Gentles A. J. Newman A. M. Liu C. L. Bratman S. V. Feng W. Kim D. (2015). The prognostic landscape of genes and infiltrating immune cells across human cancers. *Nat. Med.* 21 938–945. 10.1038/nm.3909 26193342 PMC4852857

[B14] Gingras I. Azim H. A. Jr. Ignatiadis M. Sotiriou C. (2015). Immunology and breast cancer: toward a new way of understanding breast cancer and developing novel therapeutic strategies. *Clin. Adv. Hematol. Oncol.* 13 372–382. 26352893

[B15] Goldhirsch A. Winer E. P. Coates A. S. Gelber R. D. Piccart-Gebhart M. Thürlimann B. (2013). Personalizing the treatment of women with early breast cancer: highlights of the st gallen international expert consensus on the primary therapy of early breast cancer 2013. *Ann. Oncol.* 24 2206–2223. 23917950 10.1093/annonc/mdt303 PMC3755334

[B16] Gooden M. J. M. de Bock G. H. Leffers N. Daemen T. Nijman H. W. (2011). The prognostic influence of tumor-infiltrating lymphocytes in cancer: a systematic review with meta-analysis. *Br. J. Cancer* 105 93–103. 10.1038/bjc.2011.189 21629244 PMC3137407

[B17] Harbeck N. Penault-Llorca F. Cortes J. Gnant M. Houssami N. Poortmans P. (2019). Breast cancer. *Nat. Rev. Dis. Prim.* 5:66. 10.1038/s41572-019-0111-2 31548545

[B18] Karn T. Pusztai L. Holtrich U. Iwamoto T. Shiang C. Y. Schmidt M. (2011). Homogeneous datasets of triple negative breast cancers enable the identification of novel prognostic and predictive signatures. *PLoS One* 6:e28403. 10.1371/journal.pone.0028403 22220191 PMC3248403

[B19] Li B. Severson E. Pignon J.-C. Zhao H. Li T. Novak J. (2016). Comprehensive analyses of tumor immunity: implications for cancer immunotherapy. *Genome Biol.* 17:174. 10.1186/s13059-016-1028-7 PMC4993001 27549193

[B20] Li J. Liu C. Chen Y. Gao C. Wang M. Ma X. (2019). Tumor characterization in breast cancer identifies immune-relevant gene signatures associated with prognosis. *Front. Genet.* 10:1119. 10.3389/fgene.2019.01119 31781173 PMC6861325

[B21] Liu X. S. Mardis E. R. (2017). Applications of immunogenomics to cancer. *Cell* 168 600–612. 10.1016/j.cell.2017.01.014 28187283 PMC5972371

[B22] Loi S. Sirtaine N. Piette F. Salgado R. Viale G. Van Eenoo F. (2013). Prognostic and predictive value of tumor-infiltrating lymphocytes in a phase III randomized adjuvant breast cancer trial in node-positive breast cancer comparing the addition of docetaxel to doxorubicin with doxorubicin-based chemotherapy: BIG 02-98. *J. Clin. Oncol.* 31 860–867. 10.1200/jco.2011.41.0902 23341518

[B23] Merlo A. Casalini P. Carcangiu M. L. Malventano C. Triulzi T. Menard S. (2009). FOXP3 expression and overall survival in breast cancer. *J. Clin. Oncol.* 27 1746–1752. 10.1200/jco.2008.17.9036 19255331

[B24] Savas P. Salgado R. Denkert C. Sotiriou C. Darcy P. K. Smyth M. J. (2016). Clinical relevance of host immunity in breast cancer: from TILs to the clinic. *Nat. Rev. Clin. Oncol.* 13 228–241. 10.1038/nrclinonc.2015.215 26667975

[B25] Siegel R. L. Miller K. D. Jemal A. (2019). Cancer statistics. *CA Cancer J. Clin.* 69 7–34. 30620402 10.3322/caac.21551

[B26] Sijts A. Sun Y. Janek K. Kral S. Paschen A. Schadendorf D. (2002). The role of the proteasome activator PA28 in MHC class I antigen processing. *Mol. Immunol.* 39 165–169. 10.1016/s0161-5890(02)00099-8 12200048

[B27] Sims J. E. Williams D. E. Morrissey P. J. Garka K. Foxworthe D. Price V. (2000). Molecular cloning and biological characterization of a novel murine lymphoid growth factor. *J. Exp. Med.* 192 671–680. 10.1084/jem.192.5.671 10974033 PMC2193273

[B28] Song Q. Shang J. Yang Z. Zhang L. Zhang C. Chen J. (2019). Identification of an immune signature predicting prognosis risk of patients in lung adenocarcinoma. *J. Transl. Med.* 17:70. 10.1186/s12967-019-1824-4 PMC6399972 30832680

[B29] Tang R. P. Kacinski B. Validire P. Beuvon F. Sastre X. Benoit P. (1990). Oncogene amplification correlates with dense lymphocyte infiltration in human breast cancers: a role for hematopoietic growth factor release by tumor cells? *J. Cell. Biochem.* 44 189–198. 10.1002/jcb.240440307 1980125

[B30] Underwood J. C. (1974). Lymphoreticular infiltration in human tumors: prognostic and biological implications: a review. *Br. J. Cancer* 30 538–548. 10.1038/bjc.1974.233 4614858 PMC2009334

[B31] Vano Y.-A. Petitprez F. Giraldo N. A. Fridman W. H. Sautès-Fridman C. (2018). Immune-based identification of cancer patients at high risk of progression. *Curr. Opin. Immunol.* 51 97–102. 10.1016/j.coi.2018.03.005 29554496

[B32] Wei B. Wang L. Du C. Hu G. Wang L. Jin Y. (2015). Identification of differentially expressed genes regulated by transcription factors in glioblastomas by bioinformatics analysis. *Mol. Med. Rep.* 11 2548–2554. 10.3892/mmr.2014.3094 25514975 PMC4337481

[B33] Yang S. Liu T. Nan H. Wang Y. Chen H. Zhang X. (2020). Comprehensive analysis of prognostic immune-related genes in the tumor microenvironment of cutaneous melanoma. *J. Cell. Physiol.* 235 1025–1035. 10.1002/jcp.29018 31240705

