# Supplementary figures and images for: Identification and Validation of an Immunological Expression-Based Prognostic Signature in Breast Cancer
Source: Front Genet. 2020 Sep 16;11:912. doi: 10.3389/fgene.2020.00912 (PMC7526716; doi:10.3389/fgene.2020.00912)

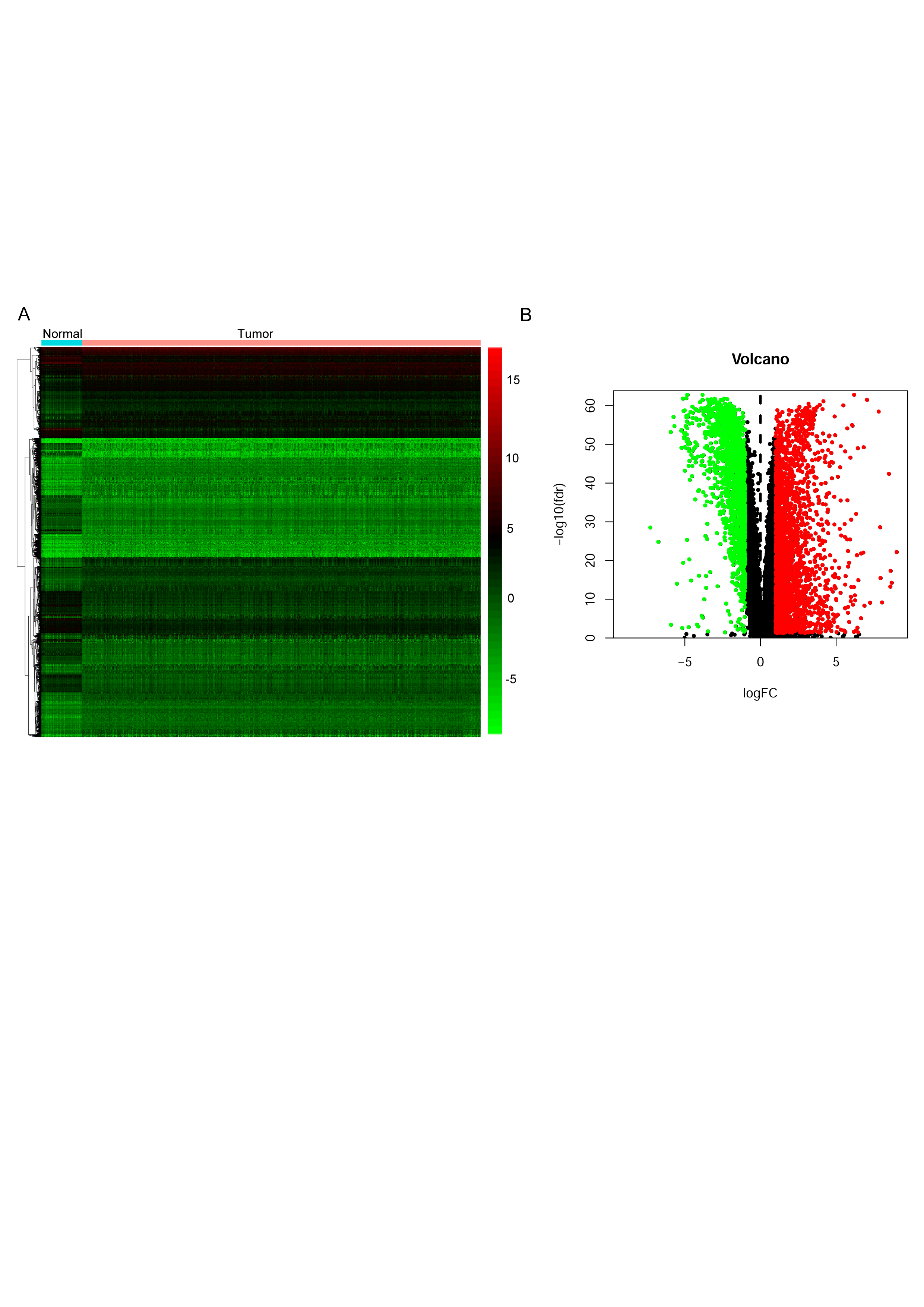

Supplement: FIGURE S1 — Heatmap (A) and volcano plot (B) of DEGs in BRCA. [file Image_1.TIF]

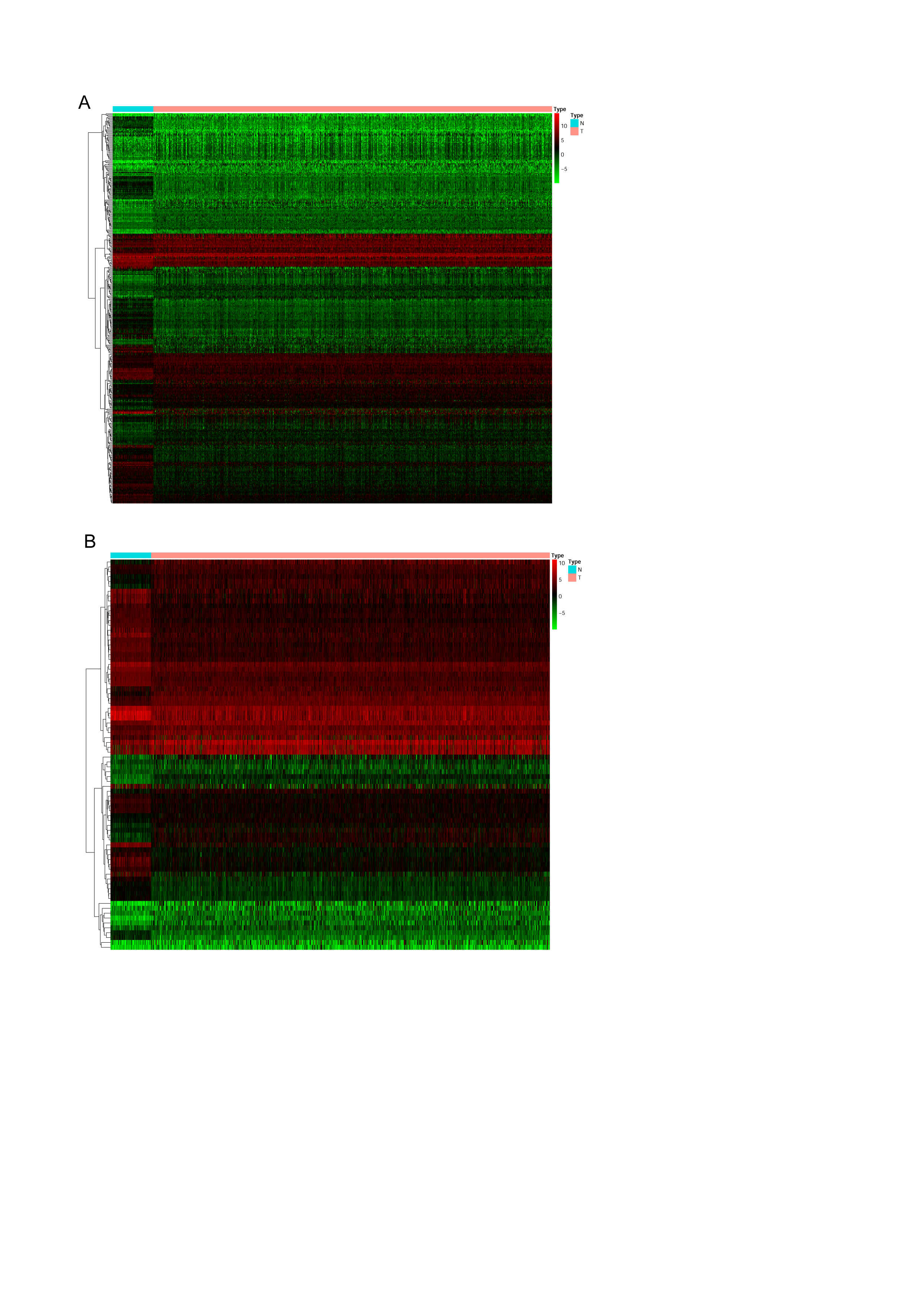

Supplement: FIGURE S2 — Heatmap of immune-related genes (A) and TFs (B) in DEGs in BRCA. [file Image_2.TIF]

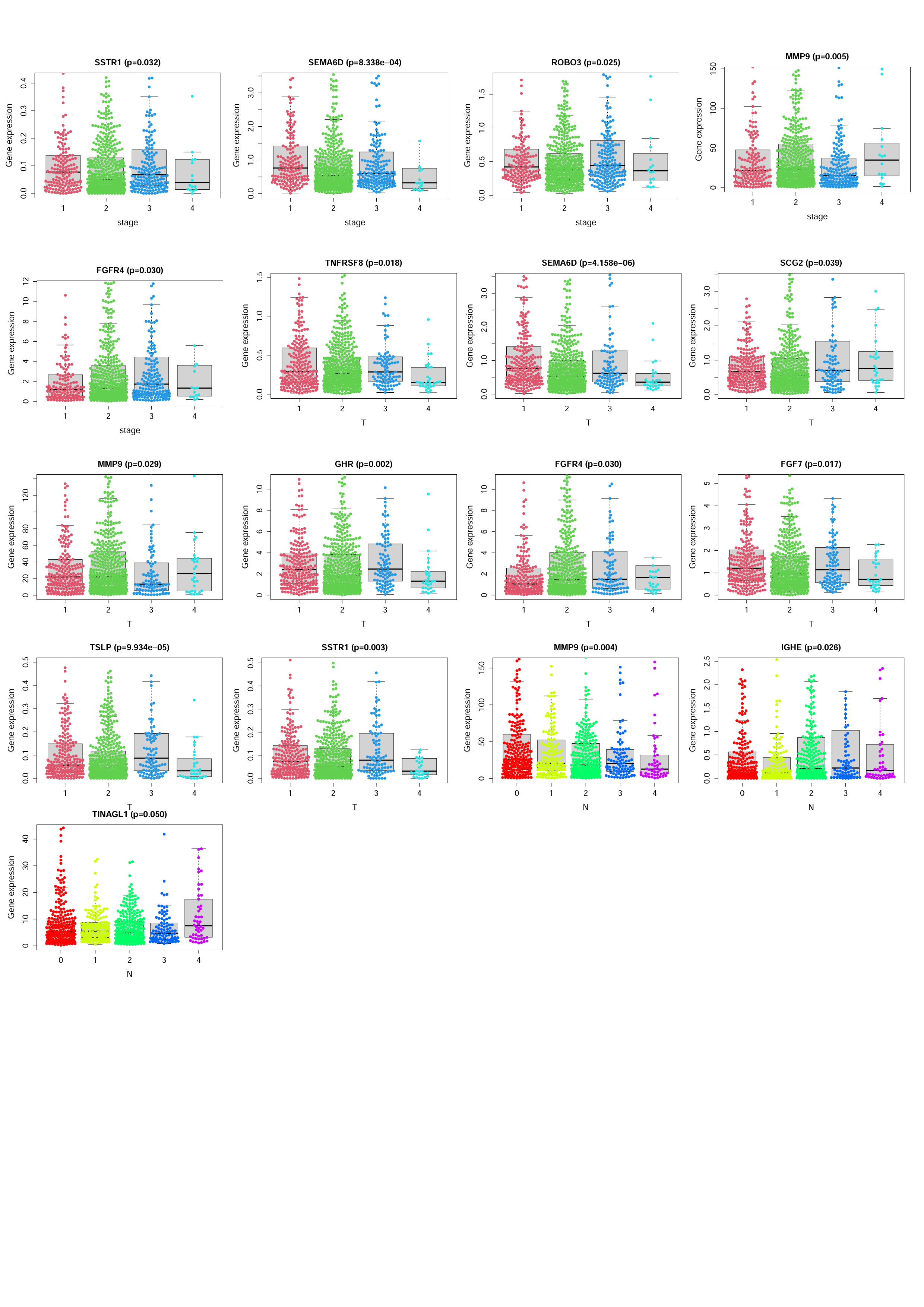

Supplement: FIGURE S3 — The correlation between expression levels of immune genes in the risk score model and clinical characteristics of BRCA patients. [file Image_3.TIF]
