# Supplementary material for: Identification and Validation of an Immunological Expression-Based Prognostic Signature in Breast Cancer
Source: Front Genet. 2020 Sep 16;11:912. doi: 10.3389/fgene.2020.00912 (PMC7526716; doi:10.3389/fgene.2020.00912)
Supplement: Supplementary file 4 [file Table_1.PDF]

Table 1 The immune-genes signature model

id	coef	HR	HR.95L	HR.95H	p value
**PSME2**	-0.01612	0.984011	0.970359	0.997856	0.023753
**TINAGL1**	0.025082	1.025399	1.011599	1.039387	0.000286
**MMP9**	0.000253	1.000253	1.000076	1.000429	0.005004
**CSRP1**	-0.03458	0.966014	0.948765	0.983576	0.000169
**ROBO3**	0.635804	1.888541	1.541644	2.313495	8.25E-10
**IGHE**	0.094539	1.099152	1.062536	1.137029	4.52E-08
**SEMA6D**	0.106281	1.112134	1.046724	1.181632	0.000589
**ADM**	0.019012	1.019194	1.002936	1.035716	0.020491
**FGF7**	0.138697	1.148776	1.079188	1.222851	1.36E-05
**SCG2**	0.001191	1.001191	0.999118	1.003269	0.26023
**TSLP**	-2.59049	0.074983	0.009463	0.594155	0.01417
**FGFR4**	0.048535	1.049732	1.030649	1.069168	2.16E-07
**GHR**	0.100568	1.105799	1.028374	1.189053	0.00662
**SSTR1**	0.070616	1.073169	1.052606	1.094133	8.43E-13
**TNFRSF8**	-1.07935	0.339816	0.173307	0.666304	0.001679
