# Supplementary material for: Identification and Validation of an Immunological Expression-Based Prognostic Signature in Breast Cancer
Source: Front Genet. 2020 Sep 16;11:912. doi: 10.3389/fgene.2020.00912 (PMC7526716; doi:10.3389/fgene.2020.00912)
Supplement: Supplementary file 5 [file Table_2.DOCX]

Supplementary Table 1: DEGs in BRCA

Supplementary Table 2: Immune-related genes from ImmPort

Supplementary Table 3: Immune-related DEGs in BRCA

Supplementary Table 4: Tumor-related TFs

Supplementary Table 5: Tumor-related TFs in DEGs in BRCA

Supplementary Table 6: Correlation coefficients between TFs and IRDEGs

Supplementary Table 7: LASSO Cox regression model in training set

Supplementary Table 8: Risk scores of each sample in training set and testing set
